# Crystallization, Luminescence and Cytocompatibility of Hexagonal Calcium Doped Terbium Phosphate Hydrate Nanoparticles

**DOI:** 10.3390/nano11020322

**Published:** 2021-01-27

**Authors:** Jaime Gómez-Morales, Raquel Fernández-Penas, Ismael Romero-Castillo, Cristóbal Verdugo-Escamilla, Duane Choquesillo-Lazarte, Annarita D’Urso, Maria Prat, Jorge Fernando Fernández-Sánchez

**Affiliations:** 1 Laboratorio de Estudios Cristalográficos, IACT, CSIC-UGR, Avda. Las Palmeras, nº 4, 18100 Granada, Spain; raquel@lec.csic.es (R.F.-P.); ismaelrc92@gmail.com (I.R.-C.); cristobal.verdugo@csic.es (C.V.-E.); duane.choquesillo@csic.es (D.C.-L.); 2 Dipartimento di Scienze della Salute, Università del Piemonte Orientale, Via Solaroli, 17, 28100 Novara, Italy; annarita.durso@uniupo.it; 3 Centro di Biotecnologie per la Ricerca Medica Applicata (BRMA), Via Solaroli 17, 28100 Novara, Italy; 4 Consorzio Interuniversitario per Biotecnologie (CIB), Località Padriciano 99, 34149 Area di Ricerca, Italy; 5 Consorzio Interuniversitario Nazionale per la Scienza e Tecnologia dei Materiali (INSTM), 28100 Novara, Italy; 6 Department of Analytical Chemistry, Faculty of Sciences, University of Granada, Avda. Fuentenueva s/n, 18071 Granada, Spain; jffernan@ugr.es

**Keywords:** terbium phosphates, calcium doped, citrate, nanoparticles, luminescence, cytocompatibility

## Abstract

Luminescent lanthanide-containing biocompatible nanosystems represent promising candidates as nanoplatforms for bioimaging applications. Herein, citrate-functionalized calcium-doped terbium phosphate hydrate nanophosphors of the rhabdophane type were prepared at different synthesis times and different Ca^2+^/Tb^3+^ ratios by a bioinspired crystallization method consisting of thermal decomplexing of Ca^2+^/Tb^3+^/citrate/phosphate/carbonate solutions. Nanoparticles were characterized by XRD, TEM, SEM, HR-TEM, FTIR, Raman, Thermogravimetry, inductively coupled plasma spectroscopy, thermoanalysis, dynamic light scattering, electrophoretic mobility, and fluorescence spectroscopy. They displayed ill-defined isometric morphologies with sizes ≤50 nm, hydration number n ~ 0.9, tailored Ca^2+^ content (0.42–8.11 wt%), and long luminescent lifetimes (800–2600 µs). Their relative luminescence intensities in solid state are neither affected by Ca^2+^, citrate content, nor by maturation time for Ca^2+^ doping concentration in solution below 0.07 M Ca^2+^. Only at this doping concentration does the maturation time strongly affect this property, decreasing it. In aqueous suspensions, neither pH nor ionic strength nor temperature affect their luminescence properties. All the nanoparticles displayed high cytocompatibility on two human carcinoma cell lines and cell viability correlated positively with the amount of doping Ca^2+^. Thus, these nanocrystals represent promising new luminescent nanoprobes for potential biomedical applications and, if coupled with targeting and therapeutic moieties, they could be effective tools for theranostics.

## 1. Introduction

Luminescent nanoparticles are excellent optical probes for uses in biological imaging since they provide the essential fluorescent contrast to analyze and study cells and tissues [1]. Different luminescent labeling agents, mainly organic dyes, and nanomaterials including semiconductor quantum dots, nanodiamonds, gold nanoparticles [2,3,4], several nanostructures labeled with organic dyes [5], and more recently calcium phosphate apatite nanoparticles (nAp) both labeled with organic dyes [6] or doped with luminescent lanthanides (Ln^3+^) ions [7,8], have been proposed for these applications. The main limitation of organic dyes is photobleaching [5], while the use of quantum dots is controversial due to their cytotoxicity [9]. In the last years lanthanide orthophosphates (LnPO_4_, Ln^3+^ = Eu^3+^, Tb^3+^, Ce^3+^, Y^3+^…) started to attract much attention because they combine the very low solubility and high thermal conductivity of the metal phosphates as host matrices with the favorable features of the luminescent lanthanide ions, such as sharp emission bands, Stokes shift of hundreds of nanometers, and luminescence lifetimes of the order of milliseconds. For all the above reasons they find applications as phosphors, catalysts, sensors and heat-resistant materials [10]. In addition, they exhibit low cytotoxicity, high photostability, and resistance to photobleaching, thus being good optical nanoprobes for bioimaging [11,12,13]. LnPO_4_·nH_2_O (*n* = 0–2) presents five polymorphic modifications, among which the low temperature hydrated phase with hexagonal structure is known as raphdophane [14,15]. In respect to Ln^3+^-doped nAp, the LnPO_4_·nH_2_O system presents the main advantage that at low temperatures (<100 °C) raphdophane is usually the only precipitated phase from aqueous solutions [15]. In calcium phosphate solutions, in contrast, several polymorphs can eventually appear including amorphous calcium phosphate (ACP), dicalcium phosphate dihydrate (DCPD, brushite), octacalcium phosphate (OCP), and Ap [16,17,18]. The synthetic methods to prepare LnPO_4_ nanocrystals include hydrothermal crystallization [19,20,21], microwave heating [22], ultrasounds [23], oil bath [24], sol-gel combined with electrospinning [25], slow crystallization using surfactants [26], or the layer-by layer adsorption followed by the reaction and crystallization at room temperature [27].

Among lanthanides, Eu^3+^ and Tb^3+^ started to be used in various host structures using different excitation wavelength in the UV region, with main emission bands in the Visible spectral region. Concerning LnPO_4_, the TbPO_4_·nH_2_O raphdophane nanorods were excited in the UV region at either 270, 312, 350 or 370 nm, and in the visible region at 482 nm, resulting in similar emission spectra, and yielding a characteristic green emission, due to ^5^D_4_–^7^F_5_ transition at 546 nm emission of Tb^3+^ [28]. Doping with Eu^3+^ allows the preparation of Eu^3+^: TbPO_4_ of hexagonal form with improved energy transfer efficiency between Tb^3+^ and Eu^3+^ and non-cytotoxicity on HeLa cells [13]. Recently, our group used citrate (cit) to prepare new luminescent cit-coated Ca^2+^:EuPO_4_·nH_2_O (with n~1) by the method of thermal decomplexing of M^(2+/3+)^/Ca^2+^/cit/phosphate/carbonate solutions (M^(2+/3+)^, metallic ions) [29]. The method was initially developed to precipitate monodisperse biomimetic apatites [30] and carbonated apatite nanocrystals, both undoped and doped with transition metals [31,32], and Eu^3+^ [8]. In this method citrate ions played an active role in the nucleation and growth steps, and remained adsorbed on the nanoparticles surface [33], a feature presented in bone apatite nanocrystals [34]. The new luminescent cit-Ca^2+^:EuPO_4_·H_2_O nanomaterials proved to be fully cytocompatible against GTL-16 human carcinoma cells and showed an improved cytocompatibility as the Ca^2+^ content increased when contacted with the more sensitive m17.ASC murine mesenchymal stem cells, thus proving to be suitable for bioimaging and cell labeling.

The aim of the present work is to employ the same bioinspired route to prepare cit- Ca^2+^:TbPO_4_·nH_2_O nanocrystals with tuned luminescence and high cytocompatibility, in the assumption that the presence of Ca^2+^ in the structure and citrate adsorbed on these luminescent solids must be beneficial from the point of view of the cytocompatibility required in bioimaging.

## 2. Materials and Methods

### 2.1. Materials and Precipitation Method

For the experiments the following reagents were purchased from Sigma-Aldrich (St Louis, MO, USA): Terbium (III) chloride anhydrous (TbCl_3_, 99.9% pure, trace metals), sodium citrate tribasic dihydrate (Na_3_(cit)·2H_2_O, where cit = citrate = C_6_H_5_O_7_, ACS reagent, ≥99.0% pure), calcium chloride dihydrate (CaCl_2_·2H_2_O, Bioxtra, ≥99.0% pure), and sodium phosphate dibasic (Na_2_HPO_4_, ACS reagent, ≥99.0% pure). Sodium carbonate monohydrate (Na_2_CO_3_·H_2_O, ACS reagent, 99.5% pure) and hydrochloric acid (HCl, ACS reagent, 37 wt % in H_2_O) were provided by Merck (Darmstadt, Germany) and Panreac (Barcelona, Spain), respectively. Solutions were prepared with ultrapure deionized water (0.22 µS, 25 °C, Milli-Q, Millipore, Burlington, MA, USA).

The synthesis of nanocrystals was carried out by the already established method of thermal decomplexing of M^(2+/3+)^/Ca^2+^/cit/phosphate/carbonate solutions [29], using Tb^3+^ as lanthanide ion (*y* = 0.09, 0.07, 0.05 and 0.03 M) and increasing concentrations of doping Ca^2+^ in the solution (*x* = 0.01 M, 0.03, 0.05, and 0.07 M) to set *x* + *y* = 0.1 M. The experiments lasted 4 h, 24 h, and 96 h. Some of them were matured for 7 days. The precipitates were subjected to washing by centrifugation with ultrapure water (6 cycles, 9000 rpm, 9 min each) and freeze-dried overnight at −50 °C under vacuum (3 mbar).

### 2.2. Physico-Chemical Characterization of Solid Nanoparticles

As-prepared powders were analyzed by different techniques, as it is described below:

X-ray powder diffraction (XRD) patterns were recorded using a diffractometer PANAlytical MPD (PANAlytical, Almelo, The Netherlands) with a Bragg-Brentano parafocusing geometry and Cu Kα radiation. Data processing of most matured samples was carried out with software TOPAS 6.0 (Coelho Software, Brisbane, Australia) [35]. The contribution of the isotropic peak broadening due to domain size was modeled using the ‘‘CS_L” TOPAS macro based on the Scherrer approximation, and considering the instrumental contribution from a measurement of LaB_6_ standard (NIST SRM 660c).

TEM observations were performed with a TEM Libra 120 Plus (EELS) instrument at 80 kV (Carl Zeiss, Jena, Germany). Prior to observation samples were dispersed in absolute ethanol (≥99.8% *v*/*v*) and deposited on copper microgrids coated with FORMVAR carbon film. HRTEM analysis were done with a TITAN G2 60-300 FEI Instrument (FEI, Hillsboro, OR, USA) operating at 300 kV, equipped with EDX Super X detector to perform microanalysis, and STEM type HAADF.

Fourier transform infrared spectra (FTIR) were recorded in transmittance mode within the wavelength range from 4000 cm^−1^ to 400 cm^−1^ using a Perkin-Elmer Spectrum One FTIR spectrometer (Perkin Elmer, Shelton, WA, USA). Pellets with ~1 wt% sample in anhydrous KBr were prepared and pressed with a hydraulic pump at 10 tons. Pure KBr pellets were used to record the background. Raman spectra were recorded with a LabRAM HR spectrometer (Jobin–Yvon, Horiba, Tokyo, Japan). The excitation line was provided by a diode laser emitting at a wavelength of 532 nm while a Peltier cooled charge–couple device (CCD) (1064 × 256 pixels) was used as a detector.

Crystal size distribution (CSD) and electrophoretic mobility (ζ-potential) were analyzed with a Zetasizer Nano ZS analyzer (Malvern Instruments Ltd., Malvern, UK) in aqueous suspensions (~0.5 mg/mL, 25 °C) contained in disposable polystyrene cuvettes. For ζ-potential versus pH measurements the MPT-2 autotitrator (Malvern Instruments Ltd., Malvern, UK) was employed to adjust the pH of the suspensions. Diluted HCl and NaOH solutions (0.25 and 0.1 M, respectively) were used as titration agents without any additional electrolyte.

Elemental analysis of Tb was carried out by inductively coupled plasma mass spectroscopy (ICP MS) using a Perkin Elmer NexION 300D ICP Mass spectrometer (Perkin Elmer). Ca and P were analyzed with Perkin Elmer ICP-OES OPTIMA 8300 spectrometer (Perkin Elmer); C and H were determined by thermoanalysis using Thermo Scientific™ FLASH 2000 CHNS/O Analyzer of Thermo Fisher Scientific (Waltham, MA, USA). TGA analyses were performed with a thermogravimetric analyzer TGA-50H model (Shimadzu, Tokyo, Japan). Samples were weighted in a platinum crucible and heated from room temperature (~28 °C) to 950 °C under 50 mL/min air flow with a heating rate of 20 °C/min.

### 2.3. Luminescence Spectroscopy

Excitation and emission spectra of both powder and aqueous suspension mg/mL samples, the latter being at ~0.5 mg/mL, were recorded using a Cary Eclipse Varian Fluorescence Spectrophotometer (Varian Australia, Mulgrave, Australia) using λ_exc_ = 375 nm, λ_em_ = 545 nm, 0.120 µs delay time (td) and 5 ms gate time (t_g_); photomultiplier voltage of 470 V and slit width_exc/em_ 5/5 nm for powder samples and photomultiplier voltage of 800 V and slit width_exc/em_ 10/10 nm for aqueous suspension particles. The excitation and emission spectra were recorded within the range 250–500 nm and 500–750 nm, respectively. Lifetime (τ) measurements were also recorded by a Cary Eclipse Varian Fluorescence Spectrophotometer (Varian Australia, Mulgrave, Australia) using λ_exc/em_ = 375/475 nm, 100 µs delay time (t_d_), 0.010 ms gate time (t_g_), photomultiplier voltage of 600 V, slit width_exc/em_ 10/10 nm and 100 cycles.

For solid sample analysis, a Cary Eclipse Solid Sample Holder (https://www.agilent.com/en/product/molecular-spectroscopy/fluorescence-spectroscopy/fluorescence-accessories/cary-eclipse-solid-sample-holder) was used to allow the measuring at the optimum angle and to minimize the effect of the thickness.

Concerning the dispersity of the measurements, they are not shown in the figures due to their lower value. In all the case the RSD (relative standard deviation) were lower than 2% (*n* = 3).

### 2.4. Cytocompatibility Tests

Nanocrystal cytocompatibility was tested on two cell lines: GTL-16 (a human gastric carcinoma cell line) and A549 (a human lung adenocarcinoma cell line). Then, 24 h after seeding cells (12,000 GTL-16 and 5000 A549 cells/well in 96-well plates), different concentrations of the differentially doped (*x* = 0.01, 0.03, 0.05, 0.07 M Ca^2+^) nanoparticles, ranging from 0.1 to 100 mg/mL, were added in 100 mL of fresh medium. Hydrogen peroxide (1 mM) was used as control of toxicity. After 72 h incubation, cell viability was evaluated by the 3-(4,5-Dimethylthiazol-2-yl)-2,5-diphenyltetrazolium bromide) (MTT, Sigma) colorimetric assay. Briefly, 20 mL of MTT solution (5 mg/mL in a PBS solution) were added to each well and the plate was incubated at 37 °C for 3 h. After the removal of the solution, 125 mL of isopropanol, 0.2 M HCl was added to dissolve formazan crystals. One hundred mL were then removed carefully, and the optical density was measured in a multiwell reader (2030 Multilabel Reader Victor TM X4, Perkin Elmer) at 570 nm. Viability of parallel cultures of untreated cells was taken as 100% viability, and values obtained from cells undergoing the different treatments were referred to this value. Experiments were performed 3 times using 3 replicates for each sample. Data were statistically analyzed and are expressed as mean ± standard deviation. Statistical analyses were performed using a one-way ANOVA with Bonferroni’s post-hoc test for grouped analyses using GraphPad Prism version 4.03 for Windows, GraphPad Software (GraphPad Prism, San Diego, CA, USA). Differences at *p* < 0.05 were considered to be statistically significant.

## 3. Results

### 3.1. Structural, Physicochemical, Morphological Characteristics, and Colloidal Stability of Solid Nanoparticles

**Figure 1:**
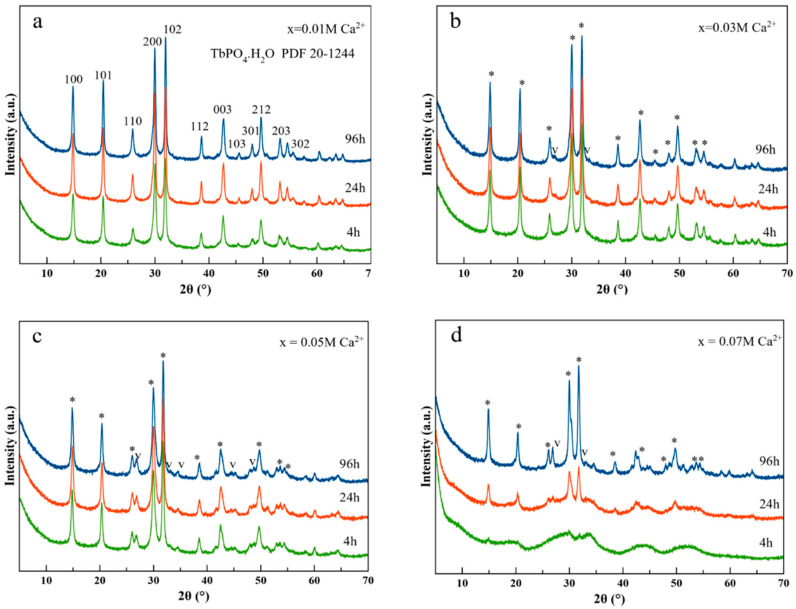
Evolution of XRD patterns with maturation time (4 h, green; 24 h, red; 96 h, blue) of samples precipitated from Ca^2+^/Tb^3+^/cit/phosphate/carbonate solutions in presence of increasing concentrations of Ca^2+^: (**a**) *x* = 0.01 M Ca^2+^; (**b**) *x* = 0.03 M Ca^2+^; (**c**) *x* = 0.05 M Ca^2+^, and (**d**) *x* = 0.07 M Ca^2+^. * hexagonal phase TbPO_4_·nH_2_O PDF 20-1244.

The XRD patterns of the solids precipitated for increasing concentration of Ca^2+^ (*x*, from 0.01 M to 0.07 M) and increasing maturation times (from 4 to 96 h) are reported in Figure 1. In particular, the patterns showing the distinguishing reflections of the hexagonal (raphdophane) phase identified as TbPO_4_·nH_2_O, space group P3_1_21 (PDF 20 1244) are displayed in panels a–d. At *x* = 0.01 M Ca^2+^ this is the only crystalline phase identified. The main reflections of this phase are located at 2θ 14.85° (100), 20.43° (101), 25.91° (110), 30.02° (200), 32.10° (102), 38.72° (112), 42.70° (003), 48.10° (301), 49.63° (212), 53.2° (203), and 54.5° (302). Other minor reflections (non-indexed) appear beyond 2θ 55°. These latter peaks are not shown in the PDF 20-1244 card, but are similar to those appearing in the PDF 20-1044 card corresponding to the raphdophane structure in the EuPO_4_·H_2_O. In addition, small differences in 2θ positions are found between the two patterns because some peak assignments in PDF file 20 1244 are affected with errors ≥ 0.2°. When x ≥ 0.03 M Ca^2+^ (Figure 1b–d), however, an additional phase started to crystallize. The reflections of this phase at ~27° and 32.8° are assigned to CaCO_3_ vaterite (PDF 33-0268), possibly doped with terbium. The presence of vaterite is more evident at *x* = 0.05 and 0.07 M Ca^2+^. Thus, this compound becomes stable in calcium-rich medium due to the presence of residual CO_3_^2−^ in the crystallizing solutions. Nevertheless, its presence does not represent a problem concerning biomedical uses since this polymorph of CaCO_3_ is biocompatible [36]. At 0.07 M Ca^2+^ we found a slightly different phase evolution with time. At 4 h, the bulging of the baseline is characteristic of an amorphous phase, probably of terbium phosphate, whereas at 24 h the reflections of the raphdophane phase started to emerge from the bulging baseline. In none of the experiments have we detected the presence of any calcium phosphate phase.

**Figure 2:**
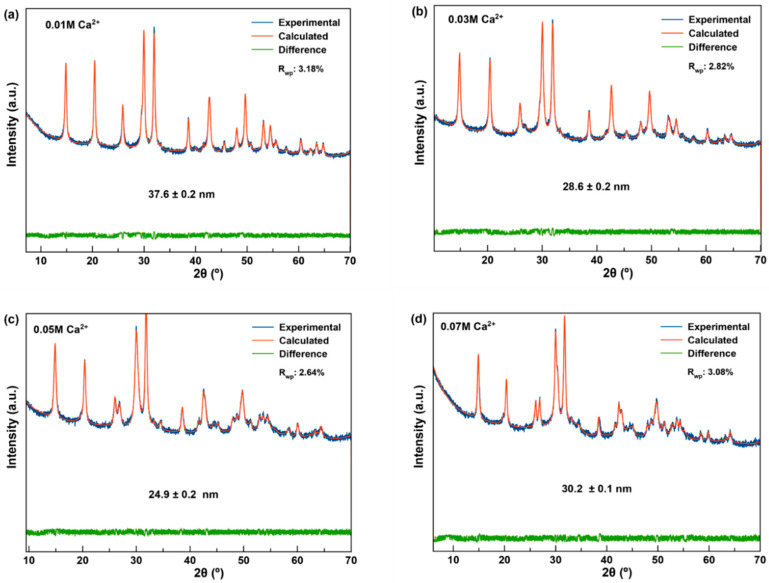
Crystalline domain sizes of Ca^2+^:TbPO_4_·nH_2_O nanoparticles prepared with, (**a**) *x* = 0.01 M; (**b**) *x* = 0.03 M; (**c**) *x* = 0.05 M and (**d**) *x* = 0.07 M Ca^2+^ for 96 h, calculated by analysis of the full XRD pattern using TOPAS 6.0. The two XRD vaterite reflections are excluded. R_wp_, weighted profile R-factor as described in Young [37].

The microstructural study of 96 h samples was performed by analysis of the full XRD pattern, excluding the reflections of vaterite (Figure 2). Results show isometric crystalline domains whose average Scherrer diameters were 37.6 ± 0.2 nm for the less Ca^2+^-doped sample (Figure 2a), 28.6 ± 0.2 nm for *x* = 0.03 M Ca^2+^ (Figure 2b), 24.9 ± 0.2 for *x* = 0.05 (Figure 2c), and 30.2 ± 0.1 for *x* = 0.07 M Ca^2+^ (Figure 2d), respectively.

**Figure 3:**
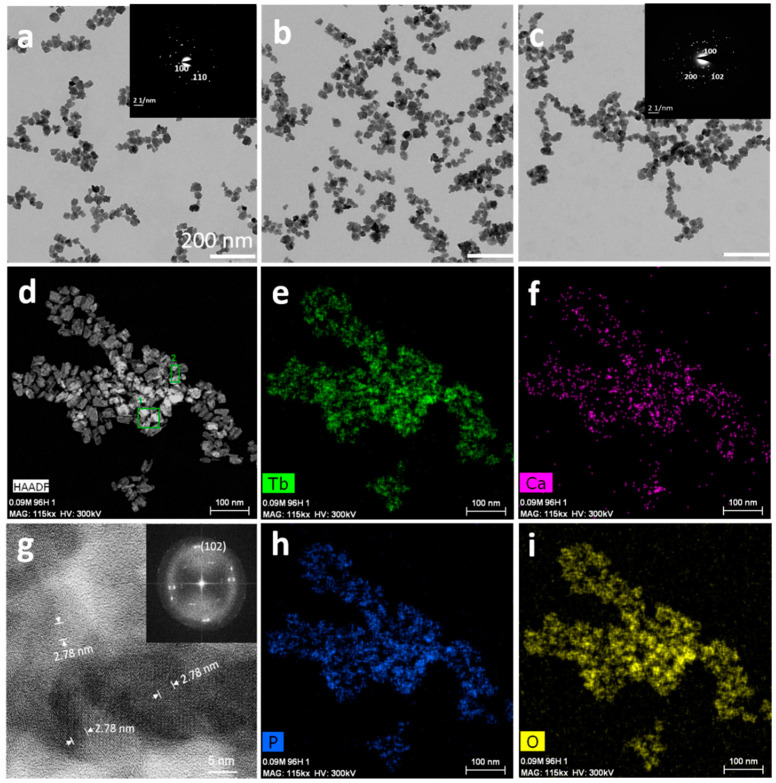
(**a**–**c**) TEM micrographs of cit-Ca^2+^:TbPO_4_·H_2_O nanorods prepared with different Ca^2+^ doping concentrations at 96 h: (**a**) *x* = 0.01 M; (**b**) *x* = 0.03 M; (**c**) *x* = 0.05 M. Insets display SAED patterns of the nanoparticles. (**d**) HAADF-STEM micrograph of sample *x* = 0.01 M Ca^2+^. (**e**,**f**,**h**,**i**) EDX element mapping analysis of Tb, Ca, P, and O in nanoparticles of image (**d**). (**g**) HR-TEM image showing lattice fringes and d-spacing of 2.78 Å corresponding to plane (102) of nanocrystals of sample *x* = 0.01 M Ca^2+^. Inset in (**g**) shows the corresponding Fourier transform (FFT) image displaying the (102) plane.

TEM observations of these precipitates show nanocrystals with ill-defined shapes and aspect ratios close to 1 (Figure 3a–c). In contrast to what has been reported for cit-Ca^2+^:EuPO_4_·H_2_O nanoparticles, no appreciable dependence of their morphology with Ca^2+^ doping was found [29].

The sizes deduced on the basis of either TEM (29 ± 5, 28 ± 3, 33 ± 6, Figure 3a–c) or SEM (*x* = 0.07 M Ca^2+^, L = 55 ± 20 nm, not shown) images are equivalent to those measured from XRD data. The indexed SAED patterns (insets) show the crystallographic planes of the hexagonal phase, i.e., (100), (110) in Figure 3a, or (100), (102), (200) in Figure 3c. The sample obtained for *x* = 0.01 Ca^2+^ was studied in more details, as shown in Figure 3d–i. Figure 3d displays the image in high angle annular dark field (HAADF) in Scanning Transmission Electron Microscopy (STEM). The elemental mappings composition of the nanoparticles (Figure 3e,f,h,i) show a homogeneous distribution of Tb, Ca, P, and O. HRTEM images of this sample (Figure 3g) reveal lattice fringes, basically monodomains, whose d-spacing of 2.78 Å corresponds to plane (102), also shown in the fast Fourier transform FFT images of this sample (Figure 3d, inset).

**Figure 4:**
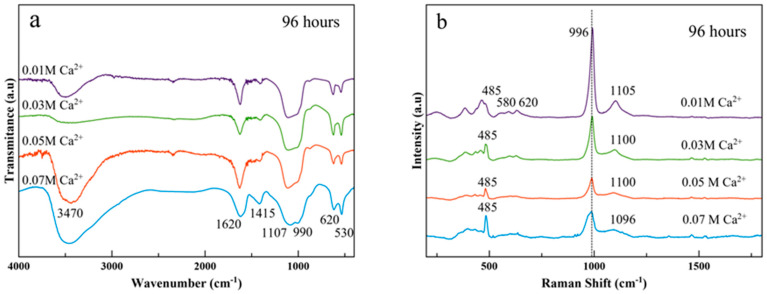
(**a**) FTIR and (**b**) Raman spectra of samples precipitated at 96 h using Ca^2+^-doping concentrations *x* = 0.01 M (purple), *x* = 0.03 M (green), *x* = 0.05 M (red), *x* = 0.07 M (light blue).

FTIR and Raman spectroscopy were employed to reveal the spectroscopic features of the Ca^2+^-doped samples (see Figure 4 for the stacking of spectra of precipitates prepared at different Ca^2+^ doping concentrations, and Supplementary Figures S1 and S2 for the stackings as a function of maturation time).

FTIR spectra of Ca^2+^-doped samples (Figure 4a) show basically the same spectroscopic features upon calcium doping, especially for *x* = 0.01, 0.03, and 0.05 M Ca^2+^. These are a broadband between 3600–3700 cm^−1^ and 2600 cm^−1^ corresponding to the O–H stretching of associated water, a main broad band at 1000–1100 cm^−1^, which corresponds to the antisymmetric stretching mode of PO_4_^3−^ groups (ν_3_PO_4_) and two intense bands at ~620 and 535 cm^−1^ ascribed to the bending mode of the same PO_4_^3−^ groups (ν_4_PO4). The broader signals around 1590–1620 cm^−1^ and 1405–1430 cm^−1^ are assignable to the antisymmetric and symmetric stretching frequencies of carboxylate (−COO^−^) groups of the adsorbed citrate [29]. No appreciable differences in peak assignments in the FTIR profiles were found after 4 h of maturation at any of the Ca^2+^ doping concentrations used (Supplementary Figure S1a–d).

In parallel, Raman characterization (Figure 4b) show the most intense band at 996 cm^−1^ (ν_1_), which can be assigned to the P–O symmetric stretching mode of PO_4_ group in raphdophane-type orthophosphates, while that at ~1100 cm^−1^ is attributed to the antisymmetric stretching (ν_3_PO_4_). The intensity of both bands decreases upon Ca^2+^ doping. The region comprised between 300 and 700 cm^−1^ corresponds to the deformation modes of the PO_4_ tetrahedron. The most intense band in this region is at 485 cm^−1^ and is assigned to the symmetric vibrations, while those at ~570 and 620 cm^−1^ can be ascribed to the asymmetric vibrations (ν_4_) [38,39]. The presence of vaterite in the spectra for *x* = 0.05 and 0.07 M Ca^2+^, with bands at 1080–1090 cm^−1^ ν_1_CO_3_ and 740–750 cm^−1^ ν_4_CO_3_ [40] is hidden by the most intense bands of the phosphate groups. Clear differences were detected when plotting Raman spectra at increasing maturation times (Supplementary Figure S2). First, an increase in the intensity of the antisymmetric stretching mode ν_3_PO_4_ at ~1100 cm^−1^, and particularly in the less Ca^2+^-doped sample (Supplementary Figure S2a) is observed. Second, the highest Ca^2+^-doped sample shows the ν_1_PO_4_ mode shifted from 970 cm^−1^ to 990 cm^−1^ (Supplementary Figure S2d), and the appearance of bands around 745 cm^−1^ and 1080 cm^−1^ (related to the vaterite phase). Also, the peaks at 845 cm^−1^ and 1460 cm^−1^ in samples at 4 and 24 h, in vibrational zones of citrate groups [41].

Thermogravimetric analyses (Supplementary Figure S3) revealed a behavior very close to the ones reported for lanthanide phosphate materials of raphdophane structure [29,42,43,44,45] but with some peculiarities reflecting the presence of citrates and carbonates in samples prepared at *x* = 0.05 and 0.07 M Ca^2+^. The first weight loss, recorded between T_amb_ and 120–140 °C of about 2 wt%, is attributed to the residual adsorbed water, while the second one between 140 °C and about 600 °C, of 5–5.5 wt%, corresponds to the release of structural water, and this is the main weight loss. The third weight loss above 600 °C is associated with the decomposition of citrate molecules in samples prepared with *x* = 0.01 and 0.03 M Ca^2+^, and with decomposition of citrate and of CaCO_3_ in CO_2_ and CaO, when *x* = 0.05 and 0.07 M Ca^2+^. Specific losses are observed in these two samples. For *x* = 0.05 M Ca^2+^ the loss of CO_2_ is around 0.6 wt% and for *x* = 0.07 M Ca^2+^ is of 2.2 wt%.

Quantitative analyses of Ca, P, Tb, C, and H of as-prepared samples are reported in Table 1. This table also shows the percent weights of structural H_2_O determined by TGA and thus, the hydration number (n) of the raphdophane phase. Percent weights of CaCO_3_ and citrate were determined by combining TGA data with mass balances. The data reveal increased percentages of dopant Ca from 0.42 wt% to 8.11 wt% in the structure of TbPO_4_.nH_2_O as the percentage of Tb decreases, according to the increase of Ca^2+^/Tb^3+^ ratio in the precursor solutions.

In these samples, the percentages of adsorbed citrate were around 1.1–1.2 wt% but in the highest Ca^2+^-doped sample that percentage rose to 4 wt%. This higher amount of citrate is likely due to the higher percentage of CaCO_3_, which can be also coated with citrate. Table 1 also shows that structural H_2_O ranges between 5.0 and 6.3 wt% rendering hydration numbers around 0.9.

**Figure 5:**
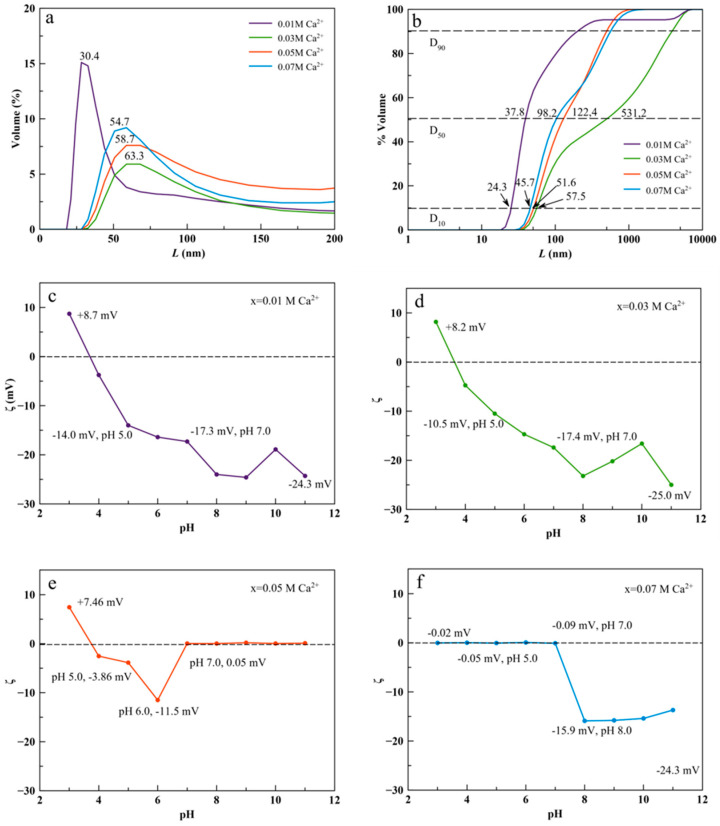
(**a**) Volume-based CSD and (**b**) cumulative volume-based CSD of cit-Ca^2+^:TbPO_4_·nH_2_O nanocrystals prepared with Ca^2+^ doping concentrations *x* = 0.01 (purple), 0.03 (green), 0.05 (red) and 0.07 M (light blue) for 96 h, measured in aqueous suspensions (pHs 8.4, 8.9, 8.3, and 7.80, respectively). (**c**–**f**) ζ-potential versus pH of aqueous suspensions of cit-Ca^2+^:TbPO_4_·nH_2_O nanocrystals.

The analysis of CSD and ζ-potential versus pH of aqueous suspensions of the nanoparticles is also relevant to assess their potential as luminescent probes in nanomedicine applications. The tendency of the colloid to disperse or aggregate in simulated physiological conditions in the blood (pH around 7.4) or in the tumor microenvironment (pH around 5–6) are related to the size and surface charge of the nanoparticles [16,46]. In addition, these features have an impact on the formation of the protein corona around the nanoparticles [47]. In this study, the CSD of cit-Ca^2+^:TbPO_4_·nH_2_O nanoparticles was plotted as cumulative volume-based distribution because they visually show the percentiles of the distribution D_10_, D_50_ and D_90_ (Figure 5 and Supplementary Figure S4), widely employed to characterize crystal populations in pharmaceutical industry. These percentiles characterize the percentage of cumulative volume undersize distribution (percentage of the population smaller than the indicated size). Thus, D_10_ is a percentile closer to the size of the individual particles while D_50_, the median of the population, is here somewhat influenced by particle aggregation. D_90_ is entirely influenced by the aggregation of nanocrystals. The percentiles for samples prepared with x = 0.01, 0.03, 0.05, and 0.07 M Ca^2+^ for 96 h were D_10_ 21, 51, 57, and 45 nm, respectively, and D_50_ 37, 531, 122, and 94 nm, respectively (Figure 5b).

In these samples, the evolution of cumulative CSD versus time (at 4 h, 24 h and 96 h, Figure S4a–d) does not follow a general trend, thus reflecting a difference in Ca composition as well as the presence of a secondary phase (vaterite) at high doping concentrations and its influence in the aggregation.

These differences are also reflected in the profile of the ζ -potential versus pH curves (Figure 5c–f). While samples obtained with *x* = 0.01 and 0.03 M Ca^2+^ displayed similar curves, with ζ -potential values −17.3 and −17.4 mV at pH 7, and −14.0 and −10.5 mV at pH 5 (Figure 5c,d), the curves of sample obtained with *x* = 0.05 M Ca^2+^ (Figure 5e) and *x* = 0.07 M Ca^2+^ (Figure 5f) show a different profile. The minimum ζ -potential values were found at pH 6.0 (−11.5 mV) and pH 8.0 (−15.9 mV) respectively. ζ -potentials were 0 beyond pH 6.0 in the first case, and below pH 8.0 in the second one.

### 3.2. Luminescence Properties of Cit-Ca^2+^:TbPO_4_·nH_2_O Nanoparticles

#### 3.2.1. Luminescence in Solid-State

It is well-known that some lanthanides, especially europium (III) and terbium (III), form highly fluorescent chelates with many different organic ligands. The sensitized fluorescence results from the ligand absorbing light, the energy of which is then transferred to the chelated metal ion. In fact, Tb(III) emits the energy as narrow-banded, line-type fluorescence with a long Stokes shift (over 250 nm) and an exceptionally long fluorescence decay time (up to 1 ms) [48]. Because of the long fluorescence decay time (over 10 times longer than the average background fluorescence) of Tb(III), a delay time (t_d_) and a gate time (t_g_) can be used during the measuring, remarkably reducing the background fluorescence.

**Figure 6:**
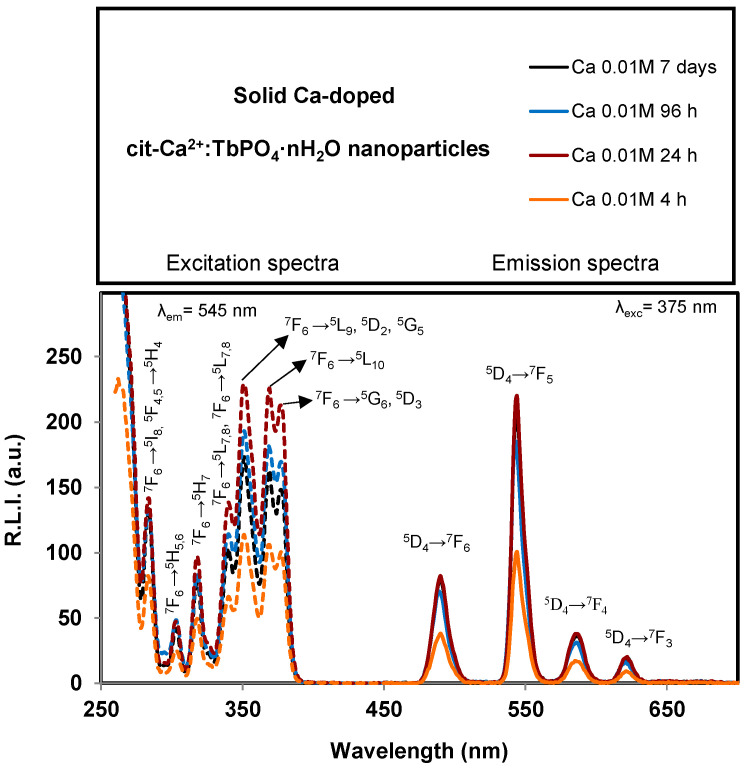
Excitation (dashed lines) and emission (solid lines) uncorrected spectra of solid cit-Ca^2+^:TbPO_4_·nH_2_O samples prepared with *x* = 0.01 M Ca^2+^ at maturation times of 4 h, 24 h, 96 h and 7 days.

The luminescence properties of solid cit-Ca^2+^:TbPO_4_·nH_2_O samples are shown in Supplementary Materials (Figures S5 and S6). They are the same as those depicted in Figure 6 which correspond to *x* = 0.01 M Ca^2+^; neither the maturation time nor the Ca^2+^ doping concentrations affect the excitation and emission wavelengths. This finding is expected because the electronic transitions of *f* orbitals are not affected by crystal’s field.

The observed excitation wavelengths for the powder were 230, 284, 300, 320, 340, 350, 368 and 375 nm. The broad bands between 200 and 300 nm, centered at 230 nm, correspond to charge transfer (called charge transfer band, CTB), which occurs by electron delocalization from the filled 2p shell of O^2−^ to the partially filled 4f shell of Tb^3+^. Also, this band can partially be attributed to the charge transfer transition X^5+^–O^2−^ [49,50]. The rest of the less intensive excitation wavelengths correspond to the ^7^F_6_ →^5^I_8_ and ^5^F_4,5_ →^5^H_4_, ^7^F_6_ →^5^H_5,6_, ^7^F_6_ →^5^H_7_, ^7^F_6_ →^5^L_7,8_ and ^7^F_6_ →^5^L_7,8_, ^7^F_6_ →^5^L_9_,^5^D_2_,^5^G_5_, ^7^F_6_ →^5^L_10_, and ^7^F_6_ →^5^G_6_, ^5^D_3_ transitions [51].

SM (see Supplementary Figure S5) shows the emission spectra of the cit-Ca^2+^:TbPO_4_·nH_2_O with *x* = 0.01 M and 96 h of maturation time using 230 and 375 nm excitation wavelengths. It is possible to conclude that the emission spectra are the same; only the emission intensity is affected. In order to increase the biological applicability of the system, 375 nm (which is closer to the visible range) was selected as excitation wavelength.

Concerning the emission wavelengths, they are centered at 490, 545, 586 and 620 nm which correspond to the Tb^3+^ ^5^D_4_→^7^F_6_, ^5^D_4_→^7^F_5_, ^5^D_4_→^7^F_4_ and ^5^D_4_→^7^F_3_ transitions, respectively [52]. The emission wavelength corresponding to the hypersensitive transition without inversion centre (^5^D_4_→^7^F_5_, 545 nm for Tb^3+^) produces the highest relative luminescence intensity (R.L.I.). Therefore, the optimum excitation and emission wavelengths of solid cit-Ca^2+^:TbPO_4_·nH_2_O samples were 375 nm and 545 nm, respectively.

Supplementary Figures S7 and S8 show the effect of maturation time (t) at different doping Ca^2+^ concentrations (*x*) on the relative luminescence intensity (R.L.I.).

The R.L.I. of samples prepared with *x* = 0.01, 0.03 and 0.05 M Ca^2+^ is not affected by the maturation time (Supplementary Figure S7). It can be due to the slowing down of the crystal growth caused by the adsorption of citrate up to 2 h maturation [29]. In contrast, for *x* = 0.07 M Ca^2+^ doping concentration, the R.L.I is highly affected; at shorter times (4 and 24 h) the signal is high, but it decreases drastically in the sample prepared at 96 h. As it was previously commented for this concentration, at shorter times (4 and 24 h) the material is amorphous without an ordered structure and with a high amount of Tb^3+^ adsorbed on its surface, showing high luminescence. However, at 96 h it is crystalline, exhibiting an improved internal ordering in which Tb^3+^ is buried in the structure of the nanoparticles, resulting in a decrease on the luminescence. It was previously reported that amorphous materials provide much more luminescence emission than crystalline ones [29]. On the other hand, this sample contains a higher proportion of citrate (~4 wt%) providing a less emissive material than at other Ca^2+^-doped concentrations.

Analyzing the variation of the R.L.I at a fixed maturation time versus *x* it is also possible to observe that the main differences are found for *x* = 0.07 M (Supplementary Figure S8). It can be explained for the same reasons commented before that, at shorter maturation times, the sample has an amorphous nature and at the higher one it is crystalline, and the amount of citrate is higher, providing a less luminescent material.

Concerning the luminescence lifetime (τ), Supplementary Figures S9–S11 show the luminescence decay curves and the variation of the luminescence lifetime versus both maturation time and Ca^2+^ doped concentrations, respectively. For each case, the decay profile was analyzed as a single exponential component (R.L.I. = A·e^(−t⁄τ) + C). It is possible to conclude that the maturation time at a given Ca^2+^ doped concentration does not affect the lifetime (Supplementary Figure S10). However, the luminesce lifetime for a given maturation time is increased by increasing the *x*. It might be due to the presence of progressive amounts of vaterite.

#### 3.2.2. Luminescence of the Nanoparticles in Aqueous Suspension

**Figure 7:**
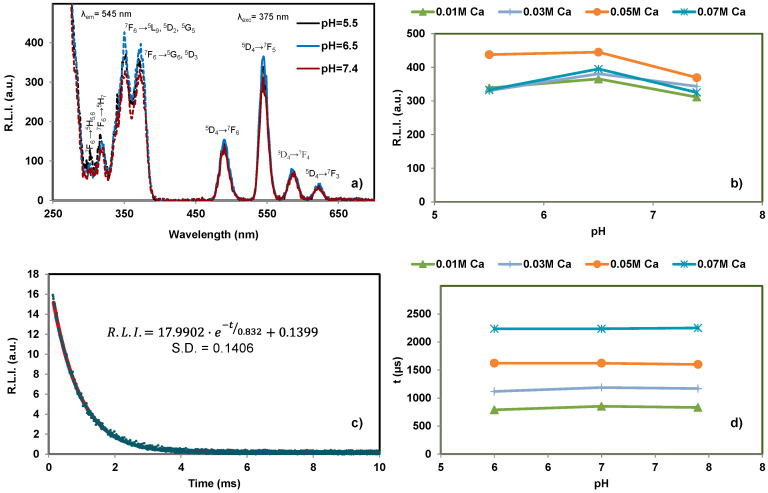
Luminescence properties of cit-Ca^2+^:TbPO_4_·nH_2_O dispersed in aqueous media with *x* = 0.01 M Ca^2+^, 96 h maturation time and 25 °C versus pH; (**a**) Excitation (dashed line) and emission (solid line) spectra; (**b**) Variation of R.L.I. versus pH; (**c**) Luminescence decay curve; and (**d**) Variation of luminescence lifetime with the pH.

The luminescence properties of cit-Ca^2+^:TbPO_4_·nH_2_O dispersed in aqueous media are similar to those depicted in Figure 7, which corresponds to *x* = 0.01 M Ca^2+^, 96 h maturation time, pH 7.4, and 25 °C. It is also possible to observe that the excitation and emission wavelength of the material dispersed in water is practically the same than those in powder as well as the luminescence lifetime is not affected by dispersing the sample in an aqueous media. On the other hand, it is possible to conclude that the material has the same luminescence properties (R.L.I. and lifetime) at any physiological pH, so variation of the pH in real biological samples does not affect the luminescence properties of the particles.

The effect of ionic strength was evaluated by suspending the particles in 0, 25, 50, 75 and 100 mM NaCl solutions (Figure S12). It is possible to deduce that neither the R.L.I. nor the luminescence lifetime is significantly affected by the ionic strength, which is also important for the final applications of these nanoparticles.

The temperature may also theoretically affect the luminescence by quenching of the excited states, i.e., for increasing T the molecular motion and collisions increase, and hence the luminescence emission decreases by increasing encounters probabilities. [53] Figure S13 shows the experimental results. To sum up, increasing from 25 to 40 °C does not affect considerably the luminescence emission of all the tested materials in suspension. The change in fluorescence intensity is normally 1% per degree Celsius [53] and the decreases for these materials were 0.2, 0.4, 2.6, and 0.6% per degree Celsius, for 0.01 M, 0.03 M, 0.05 M and 0.07 M Ca^2+^ doping concentration, respectively. This is very important in view of medical and biomedical imaging (~37.4 °C) while the rest of the experiments were performed at room temperature (25 °C).

### 3.3. Cytocompatibility of Cit-Ca^2+^:TbPO_4_·nH_2_O Nanoparticles

The cytocompatibility of cit-TbPO_4_·nH_2_O samples doped with different concentrations of Ca^2+^ was tested in a MTT assay on the GTL-16 human carcinoma cells and the A549 human lung adenocarcinoma cells, after incubation at concentrations ranging from 0.1 to 100 µg/mL. GTL-16 cells were chosen because this is the reference standard cell line used in the lab for this type of experiments. In particular, the fact that these tumor cells express a high level of receptors for a growth factor has made this cell line a good model for studying tumor cell ligand-mediated targeting by nanoparticles, possibly loaded by a drug [6,16]. A549 cells were chosen since they are easily available cells in many laboratories.

**Figure 8:**
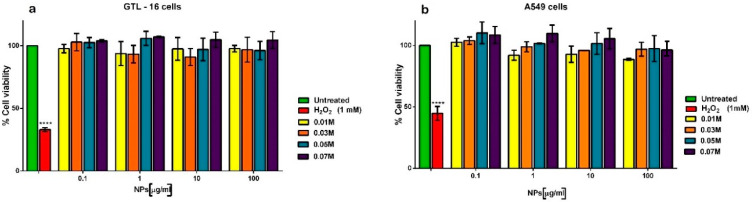
Viability of GTL-16 cells (**a**) and of A549 cells (**b**) incubated with cit-Ca^2+^:TbPO_4_·nH_2_O particles prepared with different Ca^2+^-doping concentration for three days. Viability was assessed in MTT assays. Data represent means ± SD of three independent experiments performed in triplicate and statistical analyses were carried on using One-way ANOVA, with Bonferroni comparison test. For statistical analysis all data were compared to untreated samples and only samples treated with H_2_O_2_ displayed statistically significant difference (**** *p* = 0.0005).

No toxicity was observed on both cell lines at any nanoparticle concentration (Figure 8), since in all cases a cell viability higher than 85% was observed, largely above the cut-off of 70% indicated by ISO 10993–5:2009 [54]. The presence of the doping Ca^2+^ appears to increase the biocompatibility of the nanocrystals in a dose-dependent manner. On the other side, both cell types were sensitive to the toxic activity of hydrogen peroxide, which reduced their viability to less than 50%. This in vitro assay thus shows the good cytocompatibility of the cit-Ca^2+^:TbPO_4_·nH_2_O nanoparticles.

## 4. Discussion

The above results confirm the herein reported bioinspired crystallization route succeeded in preparing cit-Ca^2+^:TbPO_4_·nH_2_O nanocrystals of the hexagonal rhabdophane phase, with n~0.9 and tailored Ca^2+^content between 0.42 wt% and 8.11 wt%. The nanocrystals displayed isometric ill-defined morphologies, and adsorbed citrate on their surfaces, a chemical feature exhibited by bone nAps [34]. As Ca^2+^ increases the risk of appearance of a secondary phase of CaCO_3_ (vaterite) must be considered. However, this trouble can be properly assumed since vaterite is biocompatible. In addition, the percent weight of citrate increases, particularly in the sample prepared with *x* = 0.07 M. As citrate molecules were not removed after different cycles of washing, they should be adsorbed not only to Ca^2+^:TbPO_4_·H_2_O but also to the CaCO_3_ surface. As hypothesized, these features have an impact on the cytocompatibility of the cit-Ca^2+^:TbPO_4_·H_2_O. In fact, all samples displayed a high cytocompatibility when tested on two human carcinoma cell lines and cell viability appears to positively correlate with the amount of doping Ca^2+^ in the nanocrystals, in line with what was reported for cit-Ca^2+^:EuPO_4_·nH_2_O nanocrystals [29].

Also important are the average sizes of the nanocrystals. The average sizes measured in TEM or SEM micrographs were equivalent to those of their crystal domains, indicating they are basically monodomains, with no appreciable dependence of their morphology (isometric) with Ca^2+^ doping. This finding contrasts with what was reported for cit-Ca^2+^:EuPO_4_·H_2_O nanoparticles [29]. Those nanoparticles showed elongated (anisometric) morphologies and their aspect ratio decreased with Ca^2+^ doping. It is worth highlighting that percentiles D_10_ of the CSD are close to these average sizes deduced from electron microscopy images. This finding indicates that aggregation is very low and D_10_ could define the nanoparticle size in the context of the whole population, particularly in the less Ca^2+^ doped sample. D_50_, instead, doubles the D_10_ percentile or is even 10 times higher in sample *x* = 0.03M Ca^2+^, suggesting a higher contribution of particle aggregation below the median size.

Concerning the ζ-potential versus pH of nanoparticle suspensions, those samples prepared at *x* = 0.01 and 0.03 M Ca^2+^ show a high and negative ζ-potential above pH 4, therefore they display great stability both at pH~7.4 simulating physiological pH, and at pHs~5–6, simulating the environment of cancer cells. The CSD of the sample prepared at *x* = 0.01 M Ca^2+^ correlates well with the ζ-potential versus pH measurements of this sample, and reflects the role of surface charge on the stability of the nanoparticles and their aggregation behavior. However, there is a lack of correlation between the CSD and the ζ-potential values for the 0.03 M Ca^2+^sample, indicating that other effects besides the nanoparticles surface charge can be involved in particle aggregation. Also, the other samples present a negative ζ-potential, but these are less negative, indicating poorer stability of the suspensions at the pHs of interest for biological applications. In the latter samples, the curves reflect the presence of vaterite, and also the increasing amount of citrate, which correlate with a relatively higher cytocompatibility. Thus, when *x* = 0.05 M Ca^2+^ the ζ -potential value close to zero indicates a high amount of surface Ca^2+^ that compensate the negative charge due to citrate at basic pH. When *x* = 0.07 M Ca^2+^ (Figure 5f) the ζ-potential values at basic pHs are again negative. This finding is due to the higher percentage of citrate negatively charged respect to positively charged surface Ca^2+^ coating both Ca^2+^:TbPO_4_·nH_2_O and CaCO_3_ particles. It was reported that the mode of adsorption of citrate on a substrate depends on the pH of the solution. Citrate molecules absorb on monodentate or bidentate configuration (i.e., on nanoapatites [55]) displaying the third carboxylate group upward toward the solution. This arrangement gives rise to a negative net surface charge that is reflected in a negative ζ -potential. While preventing aggregation, the negative ζ -potentials of nanocrystal could interfere with their interaction with the cell surface, which is also negatively charged, and their eventual uptake by cell endocytosis. This behavior, however, is not the only possibility, since some negatively charged nanoparticles, such as Fluorescein-5-isothiocyanate-labelled hydroxypatite nanoparticles [6] and magnetic nanoparticles [56], were reported to be internalized by cells.

To discuss how the nanocrystals herein described were formed, one must consider both the influence of their tridimensional crystal structure and the presence of additives in the precursor solution. Indeed, the growth morphology of a nanocrystal results from the interplay between the growth rates of its outermost crystallographic planes and the growth-inhibiting/promoting effect of the additives. The raphdophane structure of the LnPO_4_·nH_2_O is described as chains formed by lanthanide with eight-fold coordination alternating with tetrahedral orthophosphate ions. These chains are extended along with the c-axis, each one linked to four neighboring chains, thus forming open channels which are filled with between 0.5 to 2 H_2_O molecules per formula unit, stabilizing the structure [15,20,57]. Consequently, LnPO_4_·nH_2_O show the general trend to grow along [001] direction forming nanowires.

The second influence we must consider is the effect of citrate, which is a known growth inhibitor. Citrate adsorbs on specific outermost planes of incipiently formed nanocrystals slowing down its growth along the [001] direction, and leading to the formation of nanocrystals with lower aspect ratios than those formed in additive-free solutions. As we obtain basically the same crystal morphology irrespective of the amount of Ca^2+^ doping the cit-Ca+:TbPO_4_·nH_2_O structure, we conclude that Ca^2+^has limited inhibiting/promoting effect on the growth of these nanocrystals, against that of citrate, in contrast to what found in cit-Ca^2+^:EuPO_4_·nH_2_O [29]. On the other side, as already discussed above, the addition of this metal ion had a positive effect on the cytocompatibility of the nanoparticles, in line with the previous report relative to cit-Ca^2+^:EuPO_4_·nH_2_O [29]. Moreover, at the highest doping dose, Ca^2+^ enhances the adsorption of citrate, an ion contributing to biomimetic properties of apatites [34].

Finally, the luminescence study of these nanoparticles in solid phase indicate that R.L.I. is affected by neither the maturation time nor the Ca^2+^ content when *x* is lower than 0.07 M Ca^2+^, as occurred on cit- Ca^2+^:EuPO_4_·nH_2_O, while the R.L.I. at *x* = 0.07 M Ca^2+^ is high at short maturation time (due to the amorphous nature of the samples) and low at high maturation time (due to the crystalline nature of the sample and also due the higher citrate amount coating the raphdophane and vaterite nanoparticles). Concerning the luminescence properties in aqueous dispersion, neither pH nor ionic strength nor temperature affect their luminescence properties at physiological range.

According to all the above considerations the optimal conditions to synthesize cytocompatible cit-Ca^2+^:TbPO_4_·nH_2_O nanocrystals free of vaterite or with negligible amount of this phase, with enough and exploitable luminescent properties (R.L.I and luminescent lifetimes) and higher stability of their aqueous suspensions at the pH range of biological interest (5–7.4), are when using Ca^2+^ doping concentration *x* = 0.01 M and *x* = 0.03 M, at any of the maturation times from 4 to 96 h.

Summing up, all the above properties are positive relative to the applications of these nanoparticles as luminescent labeling agents and highlight the potential of the thermal decomplexing synthetic method to prepare this kind of nanophosphors.

## 5. Conclusions

Cit-Ca^2+^:TbPO_4_·nH_2_O nanocrystals of the hexagonal rhabdophane phase, with n ~ 0.9 and tailored Ca^2+^ content between 0.42 and 8.11 wt% were successfully obtained by thermal decomplexing of Ca^2+^/Tb^3+^/citrate/phosphate/carbonate solutions. These nanocrystals of about 29–37 nm are mainly monodomains and display basically isometric ill-defined morphologies, with Ca^2+^ having limited inhibiting/promoting effect on the growth of these nanoparticles, but enhancing the amount of citrate adsorbed on the surface in dose-depending manner. All the nanoparticles displayed high cytocompatibility on two human carcinoma cell lines and cell viability correlated positively with the amount of doping Ca^2+^.

Luminescence properties of the nanocrystals reveal that luminescent lifetimes increase (between ~800 and ~2600 µs) by increasing the Ca^2+^doping concentration. Their relative luminescence intensities in solid state (around 200 units) are affected by neither Ca^2+^, nor citrate content, nor maturation time for Ca^2+^ doping concentrations below 0.07 M Ca^2+^. Only at this doping concentration the maturation time strongly affects R.L.I, decreasing it. At low maturation times (4 h, 24 h) it is high (around 650 units). However, at 96 h, it strongly decreases up to ~40. This finding was related to the amorphous nature of the precipitates obtained at 4 and 24 h, whereas at 96 h, the precipitates were crystalline. In addition, this sample also contains vaterite and a higher percent weight of citrate than the samples obtained with lower doping concentrations of Ca^2+^, and therefore it presents a lower percent weight of emissive material. In aqueous suspensions, neither pH nor ionic strength nor the temperature affect their luminescence properties.

According to all the above considerations, the optimal conditions to synthesize cytocompatible cit-Ca^2+^:TbPO_4_·nH_2_O nanocrystals free of vaterite or with negligible amount of this phase, with enough and exploitable luminescent properties (R.L.I and luminescent lifetimes) and higher stability of their aqueous suspensions at the pH range of biological interest (5–7.4), are when using Ca^2+^ doping concentration *x* = 0.01 M and *x* = 0.03 M, at any of the maturation times from 4 to 96 h.

Summing up, the above properties are encouraging relative to the potential bioimaging applications of the nanocrystals. They can thus represent promising new luminescent nanoprobes for such applications and, if coupled with targeting and therapeutic moieties, they could be effective tools for theranostics.

## Figures and Tables

**Table 1:** Quantitative analyses of Ca, P, Tb, C, H, and estimated percent weights of structural citrate, H_2_O, CaCO_3_ and hydration number n of the samples prepared with Ca^2+^ doping concentration x ranging from 0.01 to 0.07 M, at 96 h.

*x* [Ca^2+^] mol/L	Ca (wt%)	P (wt%)	Tb (wt%)	C (wt%)	H (wt%)	Cit (wt%)	H_2_O str (wt%)	CaCO_3_ (wt%)	n
0.01	0.42	10.5	61.4	0.46	0.72	1.21	5.0	0	0.88
0.03	2.91	10.9	39.8	0.42	0.74	1.10	5.44	0.2	0.86
0.05	6.76, 6.09 *	12.0	27.5	0.52	0.75	1.12	6.68	0.76	0.93
0.07	10.32, 8.11 *	11.45	16.9	1.72	1.04	3.96	6.33	1.75	0.95

* wt% of dopant Ca in the TbPO_4_·nH_2_O structure.

## Supplementary Materials

The following are available online at https://www.mdpi.com/2079-4991/11/2/322/s1, Figure S1: Evolution of FTIR spectra with time of samples prepared with different Ca^2+^ doping concentrations *x* = 0.01 to 0.07 M, Figure S2: Evolution of Raman spectra with time of samples prepared with different Ca^2+^ doping concentrations *x* = 0.01 to 0.07 M, Figure S3: TGA analyses of precipitates obtained at 96 h from solutions with Ca^2+^ doping concentrations ranging from (a) *x* = 0.01 to (d) *x*= 0.07 M, Figure S4: Cumulative volume oversize distribution of the cit-Ca^2+^:TbPO_4_.nH_2_O nanocrystals prepared with Ca^2+^ doping concentrations *x* = 0.01, 0.03, 0.05 and 0.07 M at 4, 24 and 96 h, Figure S5: Excitation (dashed lines) and emission (solid lines) uncorrected spectra of solid cit-Ca^2+^:TbPO_4_·nH_2_O samples prepared with *x* = 0.01 M Ca^2+^ at maturation times of 96 h using t_d_ = 120 µs, t_d_ = 5 ms and (a) λexc/em = 230/545 nm, slit width exc/em = 2.5/2.5 nm, detector voltage 545 V; b) λexc/em = 375/545 nm, slit width exc/em = 5/5 nm, detector voltage 470 v, Figure S6: Excitation (dashed lines) and emission (solid lines) uncorrected spectra of solid cit-Ca^2+^:TbPO_4_·nH_2_O samples prepared with different Ca^2+^ doped concentration at maturation times of 4 h, 24 h, 96 h and 7 days, Figure S7: Variation of the R.L.I. of the solid cit-Ca^2+^:TbPO_4_·nH_2_O samples at the maximum excitation and emission wavelengths at several Ca^2+^ concentrations when the maturation time is changed. Figure S8: Variation of the R.L.I. of the solid cit-Ca^2+^:TbPO_4_·nH_2_O samples at the maximum excitation and emission wavelengths at several maturation time when the Ca^2+^ concentration is changed. Figure S9: Luminescence decay curve of different solid cit-Ca^2+^:TbPO_4_·nH_2_O samples at maturation times of 96 h, t_d_ = 100 µs, t_g_ = 0.01 ms, λ_exc/em_ = 375/545 nm, slit-widths_exc/em_ = 10/10 nm, and detector voltage = 600 V. Circles correspond to experimental data (100 cycles) and lines to the fitting equation. Figure S10: Variation of the luminescence lifetime of the solid cit-Ca^2+^:TbPO_4_·nH_2_O nanoparticles prepared at several Ca^2+^ concentrations when the maturation time is changed. Figure S11: Variation of the luminescence lifetime of the solid cit-Ca^2+^:TbPO_4_·nH_2_O samples at several maturation times when the Ca^2+^ concentration is changed. Figure S12: Effect of the ionic strength over the (a) R.L.I. and (b) luminescence lifetime of the cit-Ca^2+^:TbPO_4_·nH_2_O samples at 96 h maturation time dispersed in aqueous media at several Ca^2+^ concentrations. Figure S13. Effect of the temperature over the (a) R.L.I. and (b) luminescence lifetime of the cit-Ca^2+^:TbPO_4_·nH_2_O samples at 96h maturation time dispersed in aqueous media at several Ca^2+^ concentrations.
