# Supplementary material for: Crystallization, Luminescence and Cytocompatibility of Hexagonal Calcium Doped Terbium Phosphate Hydrate Nanoparticles
Source: Nanomaterials (Basel). 2021 Jan 27;11(2):322. doi: 10.3390/nano11020322 (PMC7910970; doi:10.3390/nano11020322)
Supplement: Supplementary file 1 [file nanomaterials-11-00322-s001.pdf]

Supplementary Materials:

Crystallization, Luminescence and Cytocompatibility of Hexagonal Calcium Doped Terbium Phosphate Hydrate Nanoparticles

Jaime Gómez-Morales[1,*], Raquel Fernández-Penas[1], Ismael Romero-Castillo[1], Cristóbal Verdugo-Escamilla[1], Duane Choquesillo-Lazarte[1], Annarita D'Urso[2], Maria Prat[2,3,4,5*], Jorge Fernando Fernández-Sánchez[6]

1 Laboratorio de Estudios Cristalográficos, IACT, CSIC-UGR, Avda. Las Palmeras, nº 4, E-18100 Armilla, Granada, Spain; raquel@lec.csic.es (R.F.-P.); ismaelrc92@gmail.com (I.R.-C.); cristobal.verdugo@csic.es (C.V.-E.); duane.choquesillo@csic.es (D.C.-L.)
2 Dipartimento di Scienze della Salute, Università del Piemonte Orientale, Via Solaroli, 17, 28100 Novara, Italy; annarita.durso@uniupo.it
3 Centro di Biotecnologie per la Ricerca Medica Applicata (BRMA), Via Solaroli 17, 28100 Novara, Italy
4 Consorzio Interuniversitario per Biotecnologie (CIB), Località Padriciano 99, 34149 Area di Ricerca, TS, Italy
5 Consorzio Interuniversitario Nazionale per la Scienza e Tecnologia dei Materiali (INSTM), 28100 Novara, Italy
6 Department of Analytical Chemistry, Faculty of Sciences, University of Granada, Avda. Fuentenueva s/n, 18071 Granada, Spain; jffernan@ugr.es
* Correspondence: jaime@lec.csic.es (J.G.-M.); maria.prat@med.uniupo.it (M.P.); Tel.: +34-958-2300 (J.G.-M.); +39-0321-660662 (M.P.)

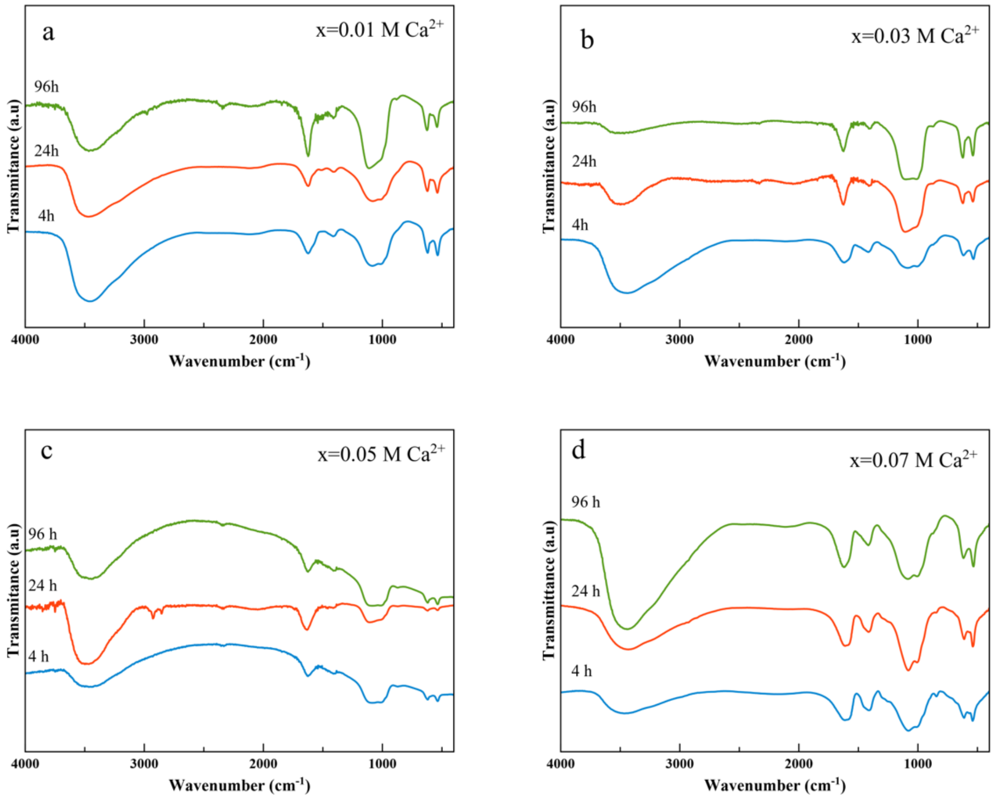

Figure S1. Evolution of FTIR spectra with time of samples prepared with different Ca^{2+} doping concentrations x = 0.01 to 0.07 M.

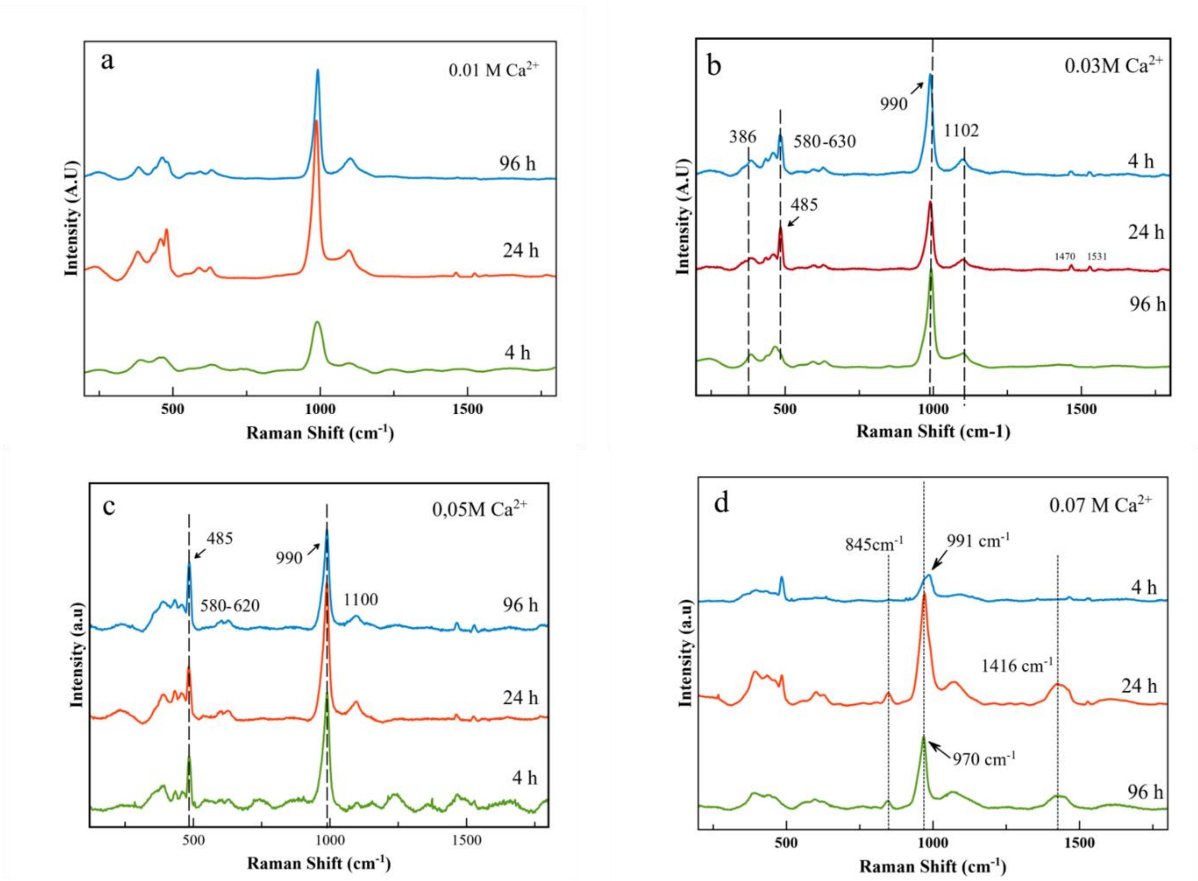

Figure S2. Evolution of Raman spectra with time of samples prepared with different Ca^{2+} doping concentrations x = 0.01 to 0.07 M.

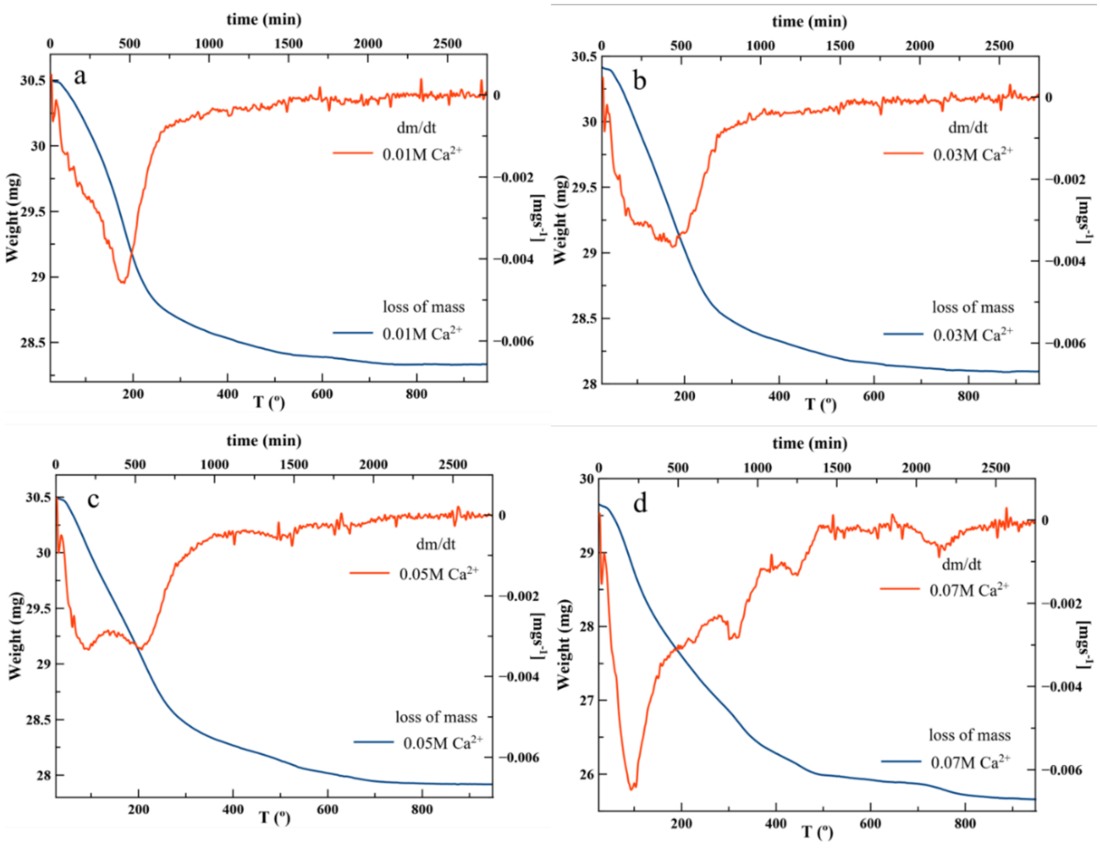

Figure S3. TGA analyses of precipitates obtained at 96 hours from solutions with Ca^{2+} doping concentrations ranging from a) x = 0.01 to d) x = 0.07 M.

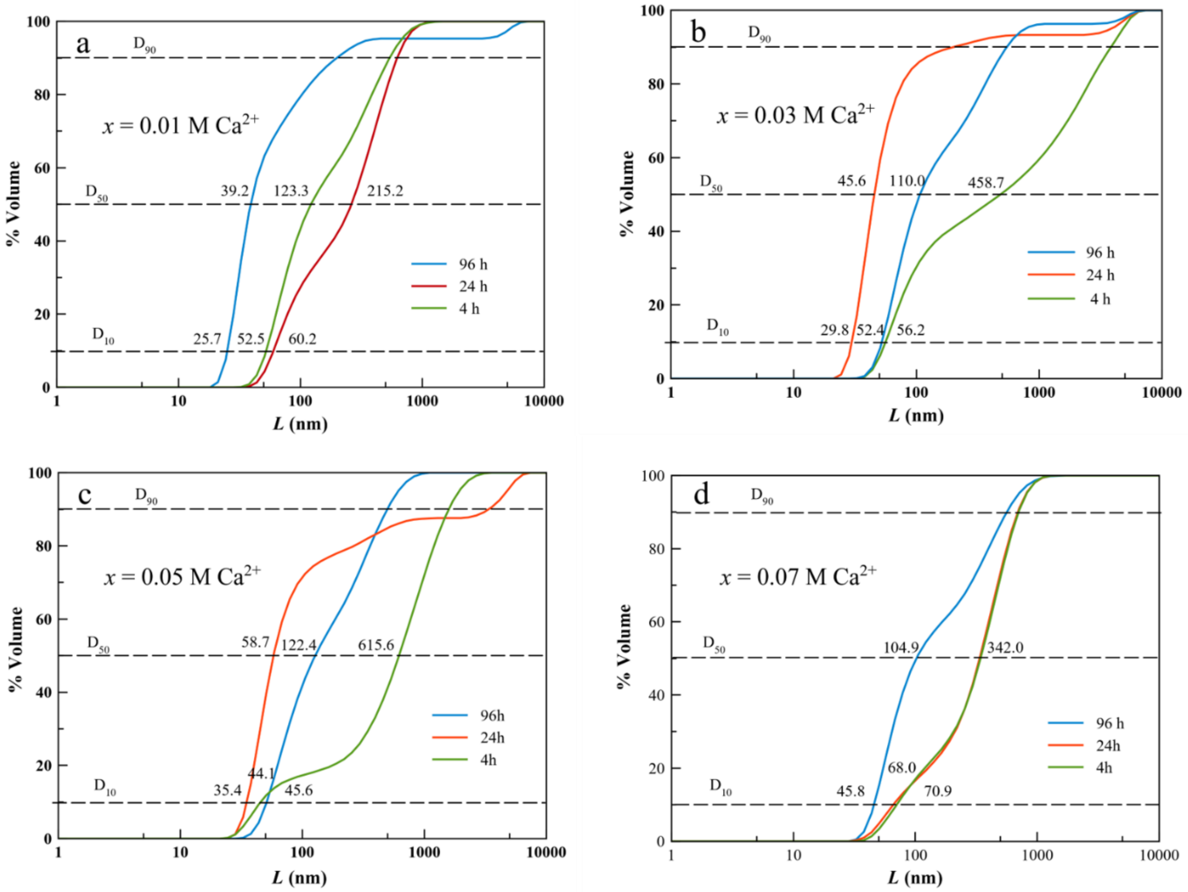

Figure S4. Cumulative volume oversize distribution of the cit-Ca^{2+}: TbPO4.nH2O nanocrystals prepared with Ca^{2+} doping concentrations x = 0.01, 0.03, 0.05 and 0.07 M at 4, 24 and 96 hours.

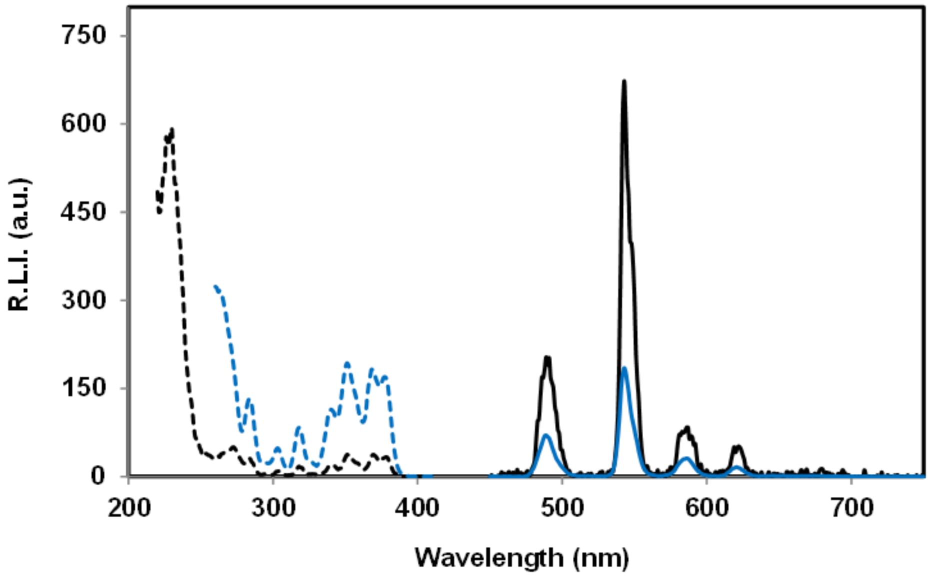

Figure S5. Excitation (dashed lines) and emission (solid lines) uncorrected spectra of solid cit-Ca^{2+}:$TbPO_4$·nH_2O samples prepared with x = 0.01 M Ca^{2+} at maturation times of 96 h using t_d = 120 μs, t_d = 5ms and a) $\lambda_{exc/em}$ = 230/545 nm, slit width $_{exc/em}$ = 2.5/2.5 nm, detector voltage 545 V for the black line; b) $\lambda_{exc/em}$ = 375/545 nm, slit width $_{exc/em}$ = 5/5 nm, detector voltage 470 V for the blue line.

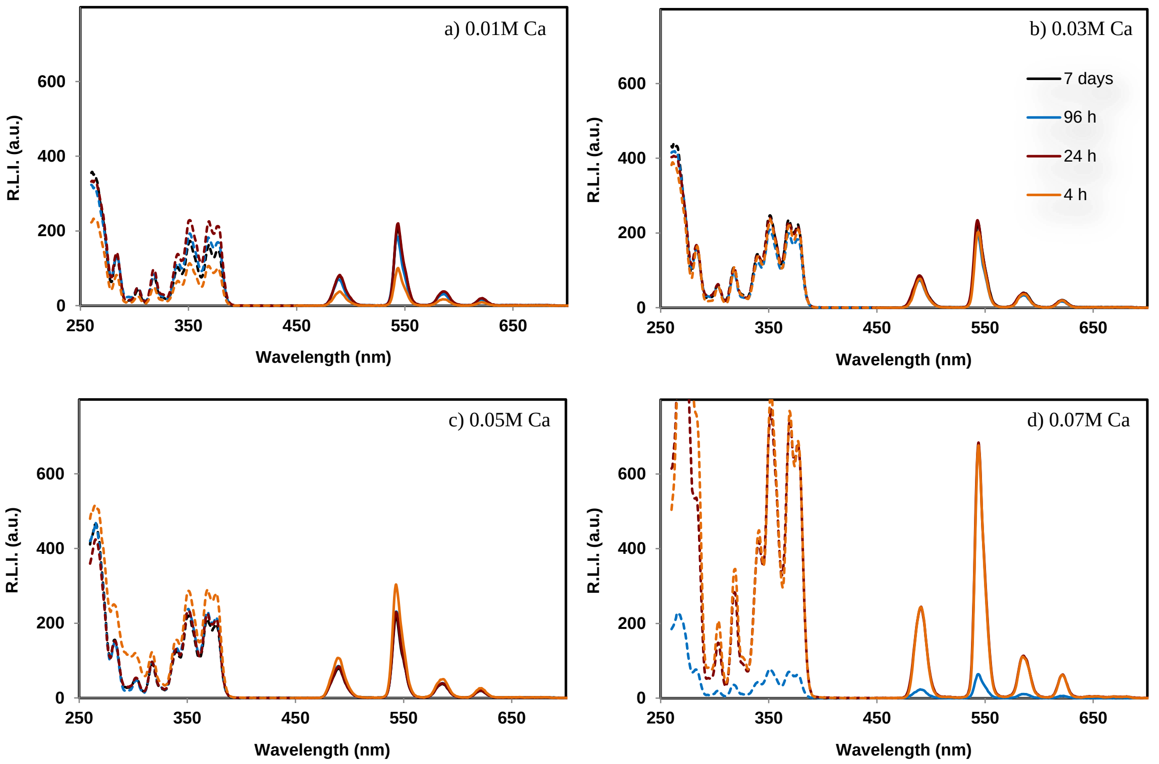

Figure S6. Excitatin (dashed lines) and emission (solid lines) uncorrected spectra of solid cit-Ca^{2+}:$TbPO_4{\cdot}nH_2O$ samples prepared with different Ca^{2+} doped concentration at maturation times of 4 h, 24 h, 96 h and 7 days.

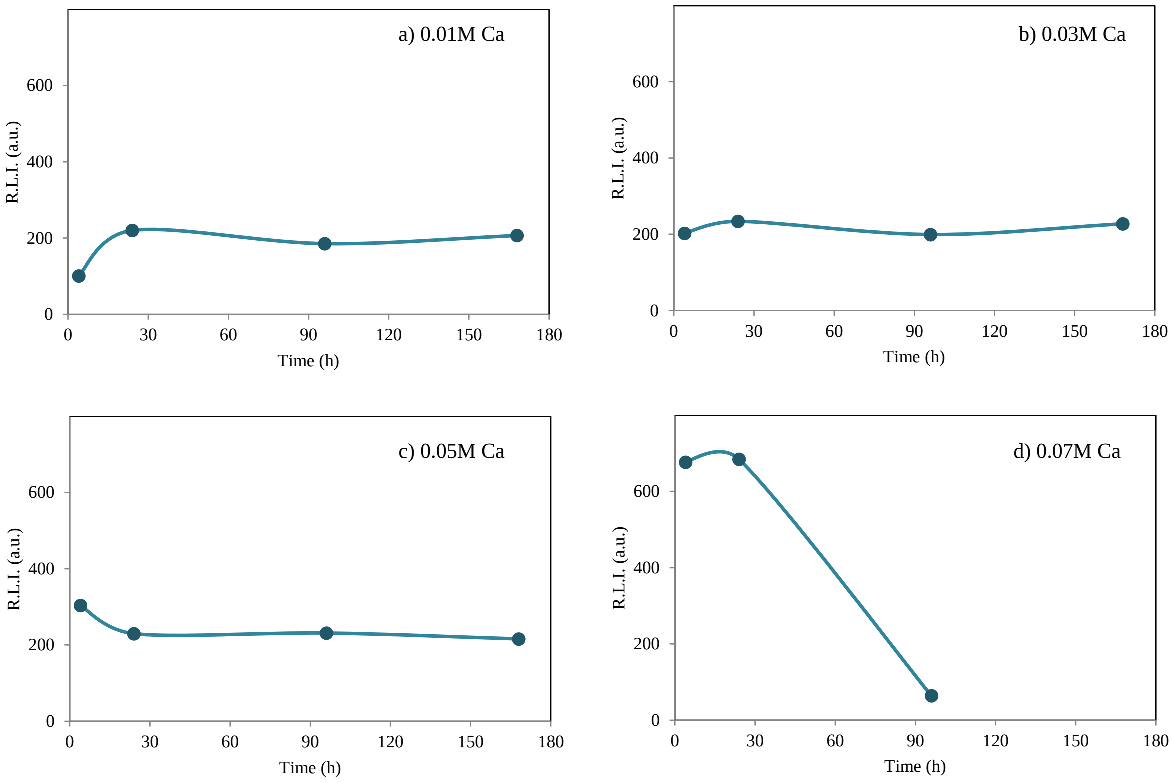

Figure S7. Variation of the R.L.I. of the solid cit-Ca^{2+}:$TbPO_4{\cdot}nH_2O$ samples at the maximum excitation and emission wavelengths at several Ca^{2+} concentrations when the maturation time is changed.

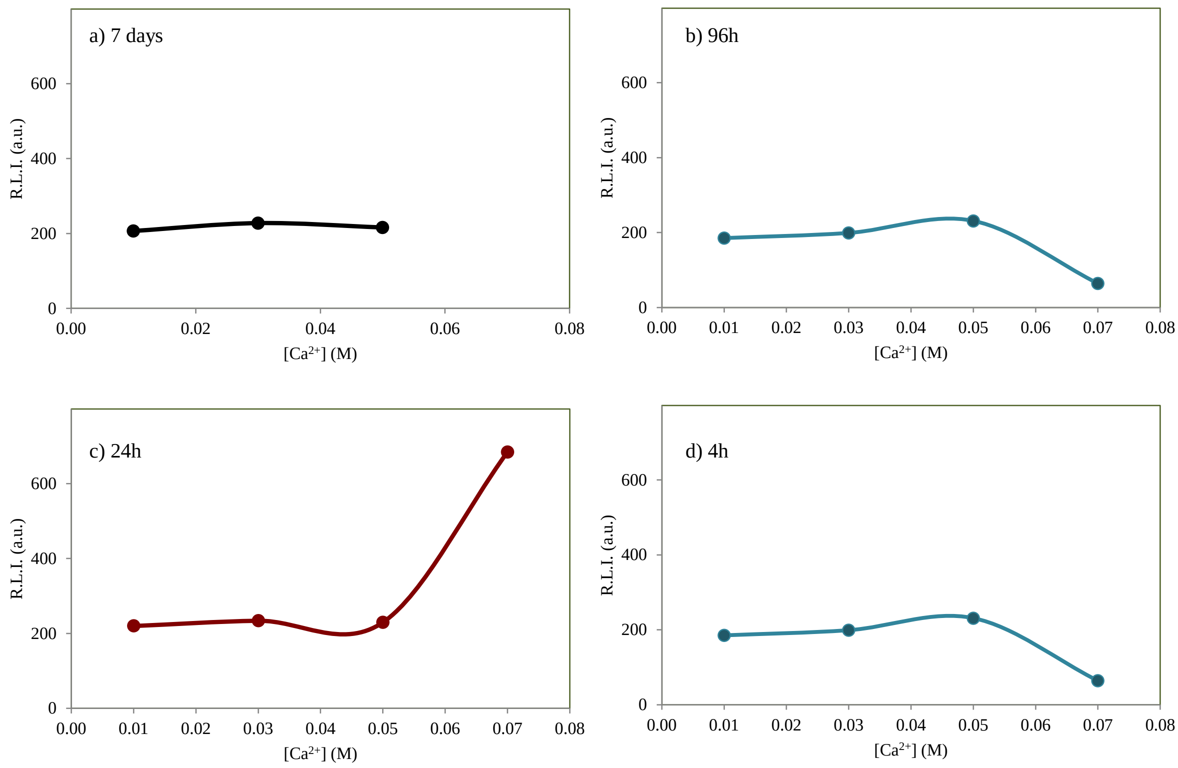

Figure S8. Variation of the R.L.I. of the solid cit-Ca^{2+}:$TbPO_4 \cdot nH_2O$ samples at the maximum excitation and emission wavelengths at several maturation time when the Ca^{2+} concentration is changed.

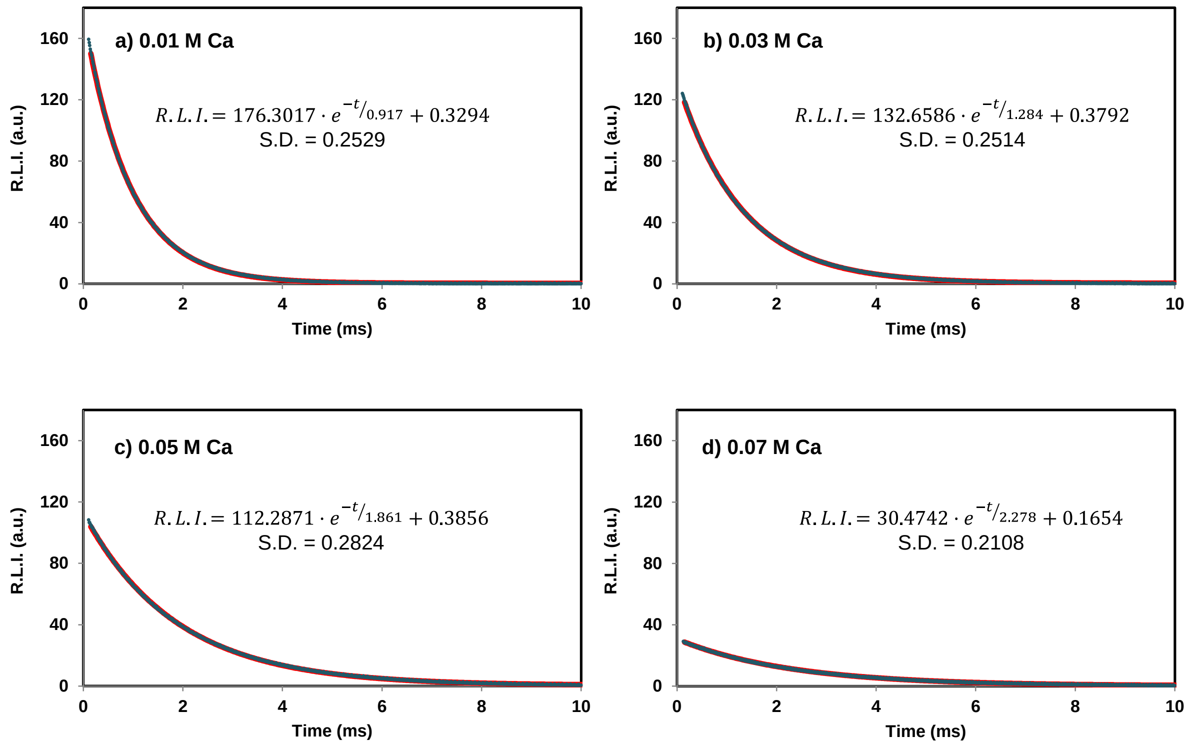

Figure S9. Luminescence decay curve of different solid cit-Ca^{2+}:$TbPO_4 \cdot nH_2O$ samples at maturation times of 96h, t_d = 100 µs, t_g = 0.01 ms, $\lambda_{exc/em}$ = 375/545 nm, slit-widths$_{exc/em}$ = 10/10 nm, and detector voltage = 600 V. Circles correspond to experimental data (100 cycles) and lines to the fitting equation.

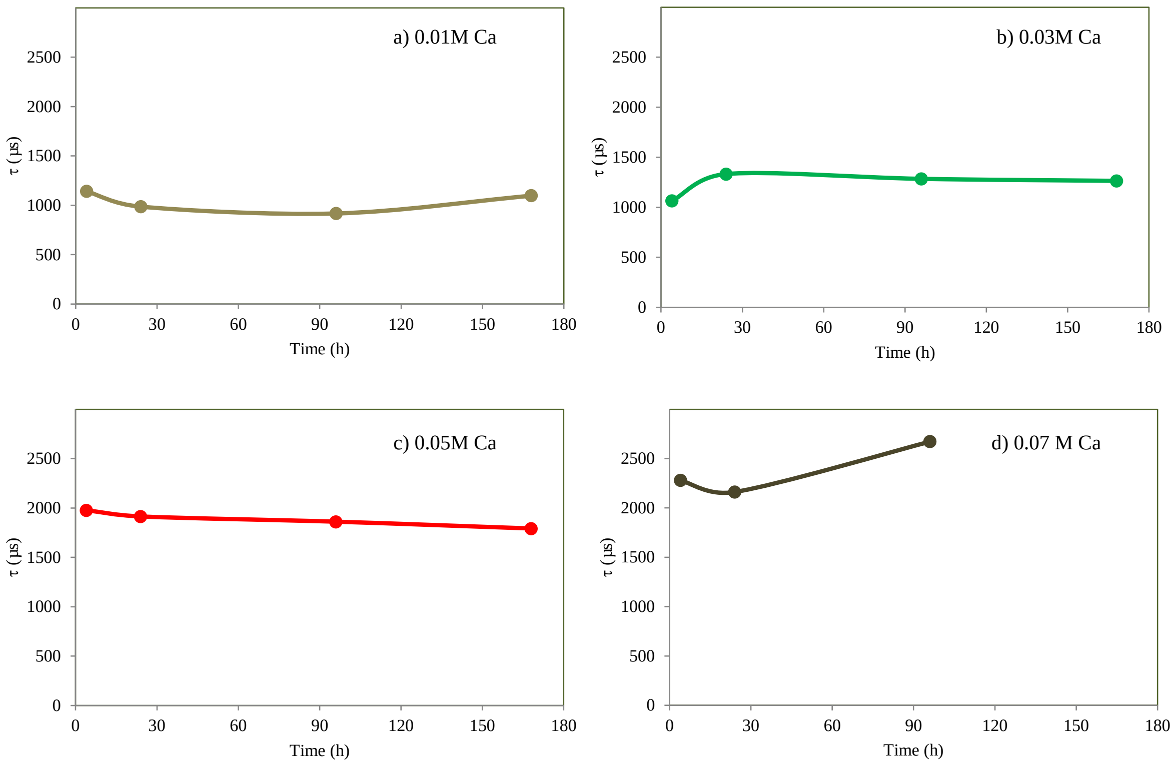

Figure S10. Variation of the luminescence lifetime of the solid cit-Ca^{2+}:$TbPO_4 \cdot nH_2O$ nanoparticles prepared at several Ca^{2+} concentrations when the maturation time is changed.

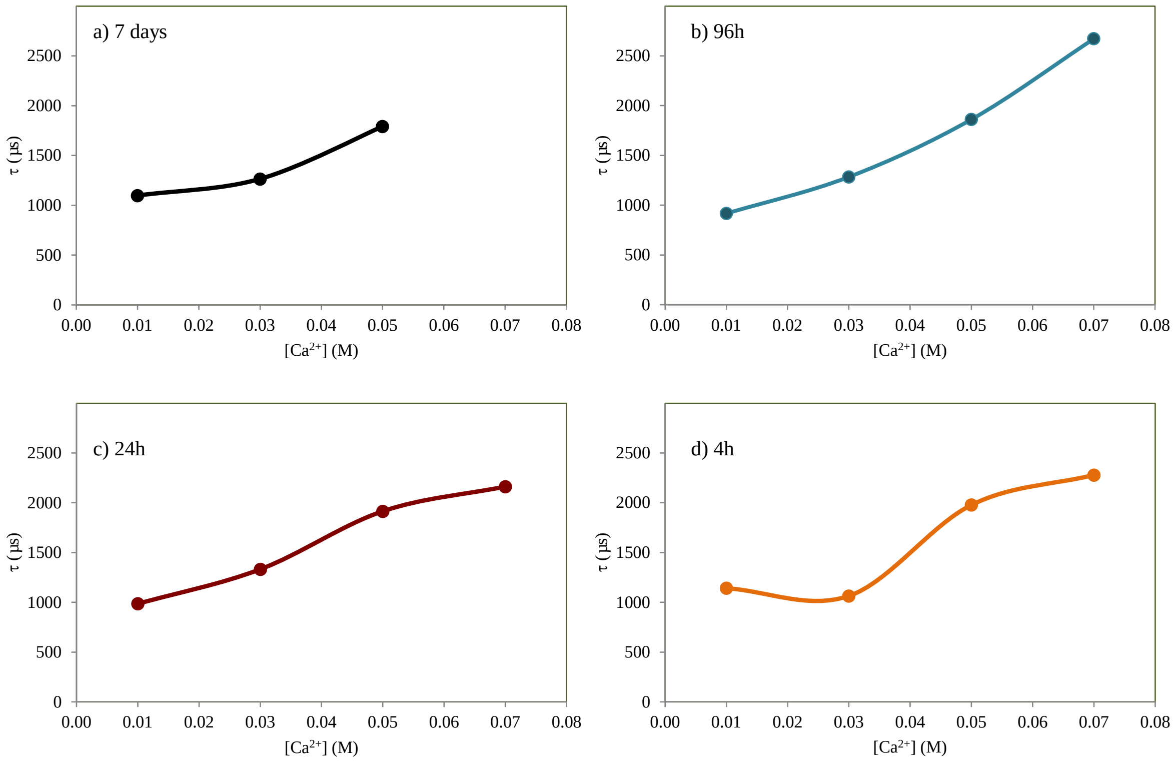

Figure S11. Variation of the luminescence lifetime of the solid cit-Ca^{2+}:$TbPO_4 \cdot nH_2O$ samples at several maturation time when the Ca^{2+} concentration is changed.

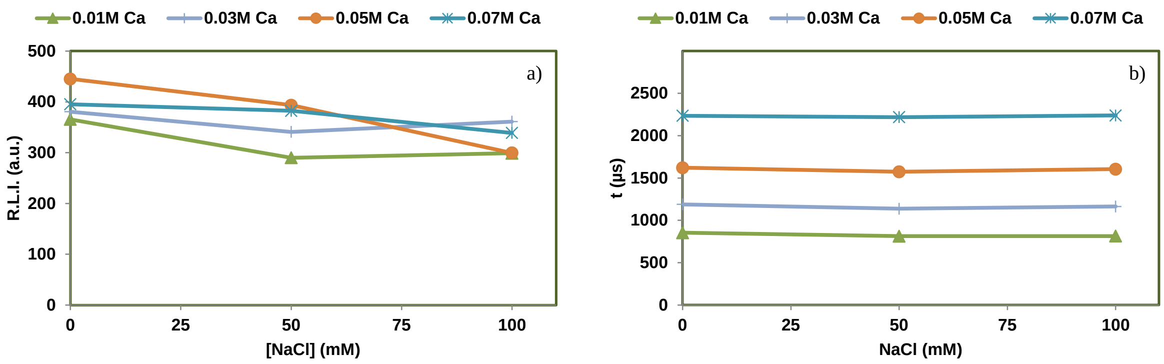

Figure S12. Effect of the ionic strength over the a) R.L.I. and b) luminescence lifetime of the cit-Ca^{2+}:$TbPO_4{\cdot}nH_2O$ samples at 96 h maturation time dispersed in aqueous media at several Ca^{2+} concentration.

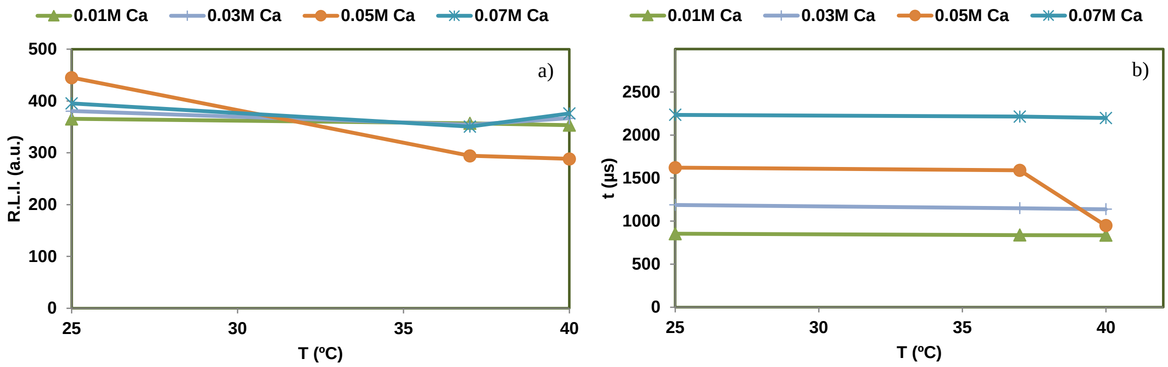

Figure F13. Effect of the temperature over the a) R.L.I. and b) luminescence lifetime of the cit-Ca^{2+}:$TbPO_4{\cdot}nH_2O$ samples at 96 h maturation time dispersed in aqueous media at several Ca^{2+} concentration is changed.
